# Deamidation alters interactions of β-crystallins in hetero-oligomers

**Published:** 2009-01-28

**Authors:** Takumi Takata, Luke G. Woodbury, Kirsten J. Lampi

**Affiliations:** 1 Department of Integrative Biosciences, School of Dentistry, Oregon Health & Science University, Portland, OR; 2 Department of Biological Sciences, Boise State University, Boise, ID

## Abstract

**Purpose:**

Cataracts are a major cause of blindness worldwide. A potential mechanism for loss of visual acuity may be due to light scattering from disruption of normal protein–protein interactions. During aging, the lens accumulates extensively deamidated crystallins. We have previously reported that deamidation in the βA3-crystallin (βA3) dimer decreased the stability of the dimer in vitro. The purpose of the present study was to investigate if deamidation altered the interaction of βA3 with other β-crystallin subunits.

**Methods:**

Deamidation was mimicked by replacing glutamines, Q85 and Q180, at the predicted interacting interface between βA3 domains with glutamic acids by site-directed mutagenesis. Human recombinant wild type βA3 or the doubly deamidated mutant βA3 Q85E/Q180E (DM βA3) were mixed with either βB1- or βB2-crystallin (βB1 or βB2) subunits. After incubation at increasing temperatures, hetero-oligomers were resolved from individual subunits and their molar masses determined by size exclusion chromatography with in line multiangle laser light scattering. Structural changes of hetero-oligomers were analyzed with fluorescence spectroscopy and blue-native PAGE.

**Results:**

Molar masses of the hetero-oligomer complexes indicated βA3 formed a polydispersed hetero-tetramer with βB1 and a mondispersed hetero-dimer with βB2. Deamidation at the interface in the βA3 dimer decreased formation of the hetero-oligomer with βB1 and further decreased formation of the hetero-dimer with βB2. During thermal-induced denaturation of the deamidated βA3 dimer, βB1 but not βB2 was able to prevent precipitation of βA3.

**Conclusions:**

Deamidation decreased formation of hetero-oligomers between β-crystallin subunits. An excess accumulation of deamidated β-crystallins in vivo may disrupt normal protein–protein interactions and diminish the stabilizing effects between them, thus, contributing to the accumulation of insoluble β-crystallins during aging and cataracts.

## Introduction

During normal aging and cataract formation, loss of visual acuity is accompanied by extensive modification of lens proteins. Numerous types of modifications have been identified and include truncation, methylation, oxidation, disulfide bond formation, advanced glycation end products, and deamidation [1-8]. These modifications change the structure and stability of lens proteins [9,10].

In the intact lens, the major structural proteins, called crystallins, are tightly packed in a very high concentration of 300–500 mg/ml [11]. However, upon processing of the lens, crystallins separate into soluble and insoluble fractions, with the insoluble proteins increasing during aging [12,13]. Modifications increase in the insoluble proteins [8,14], suggesting a potential mechanism for compromised lens function.

Of the potential post-translational modifications, deamidation is one of the major modifications in the lens and is significantly increased in aged and cataractous lenses [8]. Deamidation sites have been identified in every crystallin, and are particularly prevalent in β-crystallins [7,8,14]. For example, at least nine deamidation sites have been identified in βA3-crystallin (βA3) [8]. Furthermore, several deamidation sites are greater in the insoluble than in the soluble proteins [8,15], strongly suggesting an association between deamidation and insolubilization. The exact cause for insolubilization is not known, but may involve disruption of the normal interactions between β-crystallins.

The β-crystallins are comprised of seven polypeptide chains, βA1-, βA2-, βA3-, βA4-, βB1-, βB2-, and βB3-crystallin that when isolated in vitro form complex hetero-oligomers of dimers, βLow- (βL), and higher-ordered oligomers, βHigh-crystallin (βH) [2,3,16]. During normal maturation and aging, a hetero-oligomer intermediate in size between βL and βH increases [2] and is associated with post-translational modifications.

Two different β-crystallin dimer structures have been resolved by crystallography [17,18]. Dimers are comprised of two domains connected by a short linker peptide that is either bent as in βB1-crystallin (βB1) or extended as in βB2-crystallin (βB2). Each domain has two Greek key motifs comprised of β-strands. This interface has been well characterized, particularly in βB2, and hydrophobic interactions with salt bridges are thought to stabilize the interacting interface of the dimer [19-21]. Similar interactions most likely occur between different β-subunits. βB1 and βB2 have been reported to make hetero-oligomers with βA3 in vitro [22,23].

We have previously reported the effects of deamidation at the critical interacting interface in β-crystallins [10,24-28]. Stability was significantly decreased due to deamidation, without the homo-dimer dissociating. Deamidation introduces a negative charge at physiologic pH and most likely created a cavity or space at the interface that contributed to the structural changes that decreased stability [21]. The purpose of this study was to investigate the effect of deamidation at the interface in βA3 on subunit-subunit interactions with other β-subunits to mimic the more complex interactions found in vivo.

## Methods

### Expression and purification of recombinant protein

Wild type (WT) human β-crystallins, βB1, βB2, and βA3, were recombinantly expressed in *E. coli* as described previously [24,26,27]. Deamidation was mimicked by replacing glutamines with glutamic acids, using site directed mutagenesis to generate the Q85E/Q180E βA3 (DM βA3) mutant as previously reported [27] (Quick Change Mutagenesis, Stratagene, Cedar Creek, TX).

All human recombinant β-crystallins were purified by successive ion-exchange chromatography [10,27,28] and the purity of the proteins was checked by SDS–PAGE and mass spectrometry. Proteins were flash frozen in liquid nitrogen and stored at −80 °C stock without lyophilization. Human αA-crystallin was expressed and purified as previously described [29].

### Size exclusion chromatography with in line multi angle laser light scattering (SEC-MALS)

To investigate the effects of deamidation on the formation of hetero-oligomers as a function of temperature, a one to one molar ratio of recombinant WT βA3 or DM βA3 was premixed with either βB1 or βB2 at a final concentration of 0.4–0.5 mg/ml followed by incubation for 90 min at 37 °C or 55 °C. Samples were equilibrated in buffer (pH 6.8) containing 29 mM Na_2_HPO_4_, 29 mM NaH_2_PO_4_, 100 mM KCl, 1 mM EDTA, and 1 mM DTT. The mixture was filtered and injected onto a SEC-MALS system with in-line refractive index detector (HELEOS and Opti-Lab instruments; Wyatt Technology Inc. Santa Barbara, CA). Molar masses were calculated from the intensity of scattered light at 18 different angles according to Rayleigh light scattering principles and using software provided by the manufacturer. The protein concentration was determined from the refractive index or UV detector and a dn/dc value of 0.185 was used [30].

To investigate the effects of deamidation on the formation of hetero-oligomers as a function of time at physiologic temperature, mixtures prepared as described above were incubated for 30, 60, 90, 120 and 300 min at 37 °C. Additionally, a mixture of a 5:1 molar ratio of βB1 to βA3 was incubated for 30 min, to assure a concentration favoring dimer formation of βB1 [10]. Concentrations of individual proteins were calculated using the UV absorbance at 280 nm and extinction coefficients of 2.07 (mg/ml)^−1^cm^−1^ for βB1, 1.71 (mg/ml)^−1^cm^−1^ for βB2, and 2.62 (mg/ml)^−1^cm^−1^ for βA3. Samples (100 μl) were injected onto a Superose 12 10/300 GL column (Amersham Biosciences Corp., Piscataway, NJ) equilibrated in the same buffer with a flow rate of 0.2 ml/min.

### Circular dichroism and fluorescence of hetero-oligomers

The eluted peaks from SEC-MALS containing hetero-oligomers were pooled and concentrated to 1.0 mg/ml, then exhaustively dialyzed into 5 mM NaH_2_PO_4_ and 5 mM Na_2_HPO_4_ (pH 6.8), containing 100 mM NaF for analysis by circular dichroism (CD). Measurements were obtained using a JASCO J-810 spectropolarimeter (JASCO, Easton, MD). Hetero-oligomer samples were measured at 0.15 mg/ml in a 0.1 cm cell for far-UV scans. Experiments were repeated on two different protein preparations. Concentrations of hetero-oligomers were determined by the BCA assay (Pierce, Rockford, IL) and by amino acid analysis (Molecular Structure Facility, UC Davis, Davis, CA).

The tertiary structures of hetero-oligomers were determined by fluorescence spectrometry. The fluorescence buffer (pH 7.0) contained 50 mM Na_2_HPO_4_, 50 mM NaH_2_PO_4_, 5 mM DTT, and 2 mM EDTA. Proteins at 1 μM were incubated for 24 h at 22 °C, and measured on a Photon Technology International QM-2000–7 spectrometer using the manufacture’s supplied software, FeliX (Photon Technology International, Lawrenceville, NJ). Emission spectra were recorded between 300 and 400 nm with an excitation wavelength at 283 nm or 293 nm. Slit widths were set to 2 nm. Emission spectra were corrected for the buffer signal.

### Shape determination by blue-native-electrophoresis (BN-PAGE)

To investigate the shape of each hetero-oligomer, the same samples analyzed by SEC-MALS were analyzed by BN-PAGE. Samples were mixed in a 1:1 ratio (v/v) of sample buffer (141 mM Tris-HCl, 106 mM Tris-Base, 0.5 mM EDTA, 15% [v/v] glycerol, 0.002% Bromophenol blue at pH 7.0). Blue-native electrophoresis was performed as previously described with some modifications using pre-cast, 1.0 mm thick 8×8 cm, polyacrylamide Nu-PAGE 4%–12% Bis-Tris gels (Invitrogen, Carlsbad, CA). Anode buffer was 50 mM Bis-Tris (pH 7.0) and cathode buffer was 15 mM Bis-tris, 50 mM Tricine, 0.02% coomassie brilliant blue-G250 (pH 7.0). BN-PAGE was performed at 200 V and 4 °C until the blue dye reached the bottom of the gel. Proteins were visualized with SimplyBlue SafeStain (Invitrogen) after overnight washing with water.

### Heat induced precipitation

Heat induced precipitation of complex β-crystallin was measured in a thermal jacketed cuvette with constant stirring (Cary 4 Bio UV-Visible spectrophotometer; Varian). Heat incubation was performed in the same buffer as the SEC-MALS experiments described above. All samples at 1:1 molar ratios were concentrated to a final total concentration of 0.1 mg/ml. The turbidity of the proteins, as an indicator of aggregation and precipitation was monitored at 405 nm. All samples were added to preheated buffer in the cuvette and incubated at 55 °C for 180 min.

## Results

### Homo-oligomerization of β-crystallins

**Figure 1:**
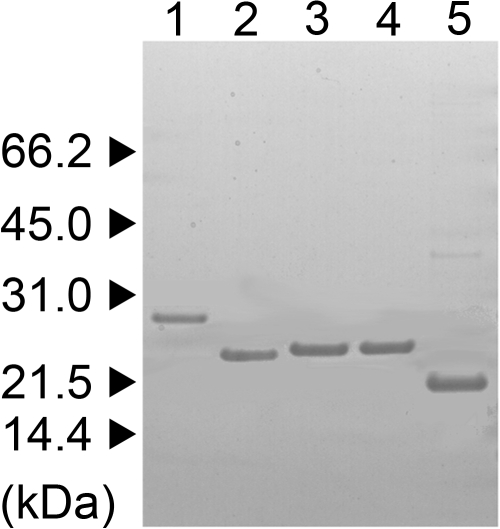
Purified recombinant crystallins. Recombinantly expressed proteins were purified to near homogeneity as indicated by a single band on SDS-PAGE. Each protein (1 μg) was visualized with Coomassie blue stain on a 1.0 mm thick, 4-12 % Bis/Tris gel. Proteins were βB1 (lane 1), βB2 (lane 2), βA3 WT (lane 3), βA3 DM (lane 4) and αA-crystallin (lane 5). Triangles indicate positions of molecular weight markers.

**Figure 2:**
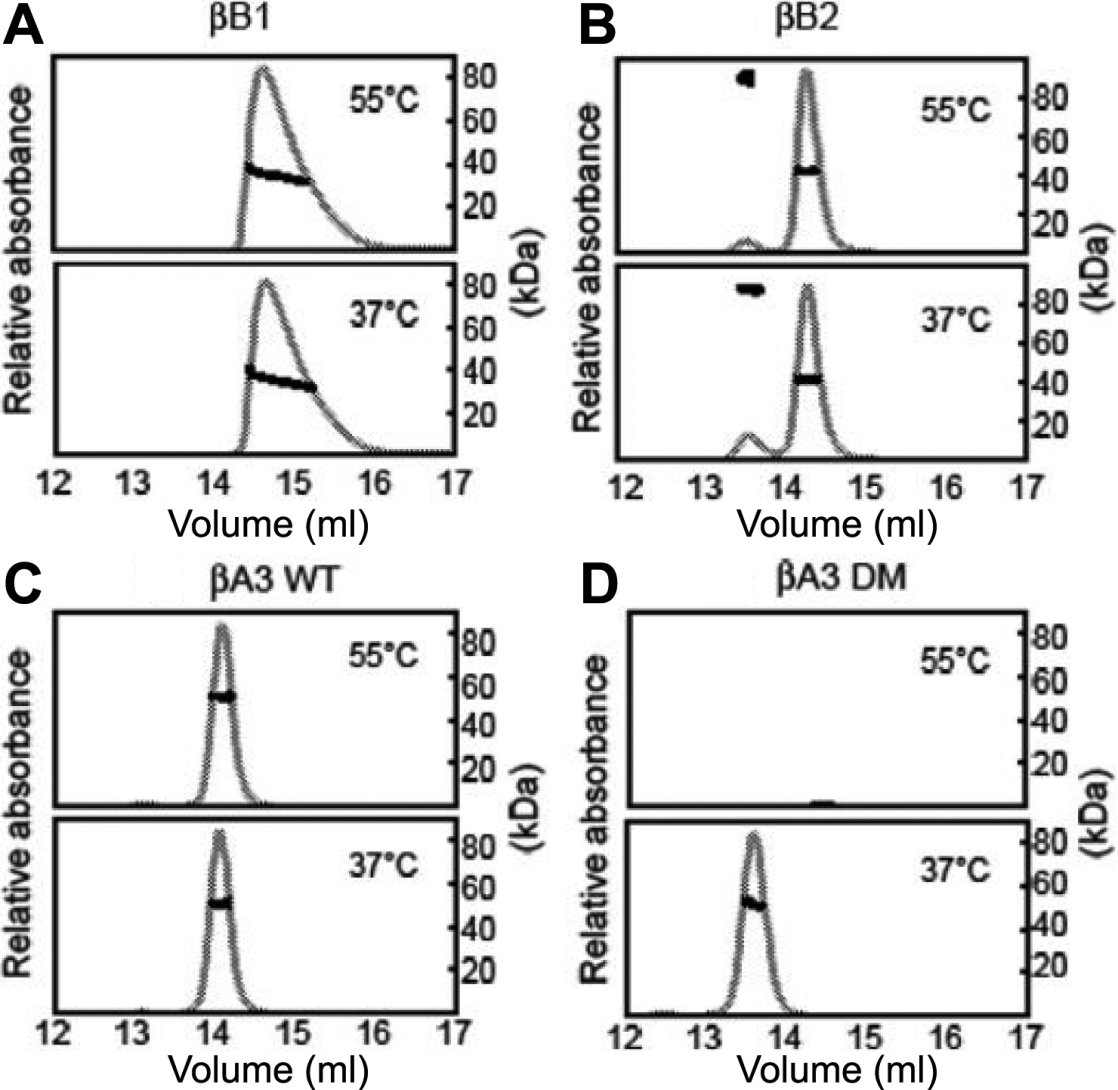
Homo-oligomerization of β-crystallins. Purified crystallins were incubated for 90 min at either 37 °C or 55 °C and then subjected to SEC-MALS. Eluting proteins were detected by UV absorption at 280 nm (grey line). Molar masses were determined across the peak (solid squares). From molar masses, βB1 was a mixture of monomer-dimers (**A**); βB2 was predominantly a dimer with less of an earlier eluting peak (**B**); βA3 WT was a dimer (**C**); and βA3 DM was a dimer at 37 °C, but had precipitated at 55 °C (**D**).

Recombinantly expressed crystallins were purified to greater than 95% purity indicated by a single band on SDS–PAGE (Figure 1). βB1, βB2, βA3, and DM βA3 were incubated for 90 min at 37 °C or 55 °C, and subjected to SEC-MALS (Figure 2A-D). All samples eluted in a single peak with βB2 having an additional earlier eluting peak. βB1 eluted as a monomer-dimer mixture. βB2 eluted mostly as a dimer with a lesser amount of a tetramer, and WT βA3 and DM βA3 eluted as dimers. βB2 and WT βA3 eluted as dimers at both temperatures. A DM βA3 dimer was not observed at 55 °C by SEC-MALS, due to precipitate forming that would have been filtered immediately before SEC-MALS (Figure 2D and [27]).

### Hetero-oligomerization of βA3 with βB1

**Figure 3:**
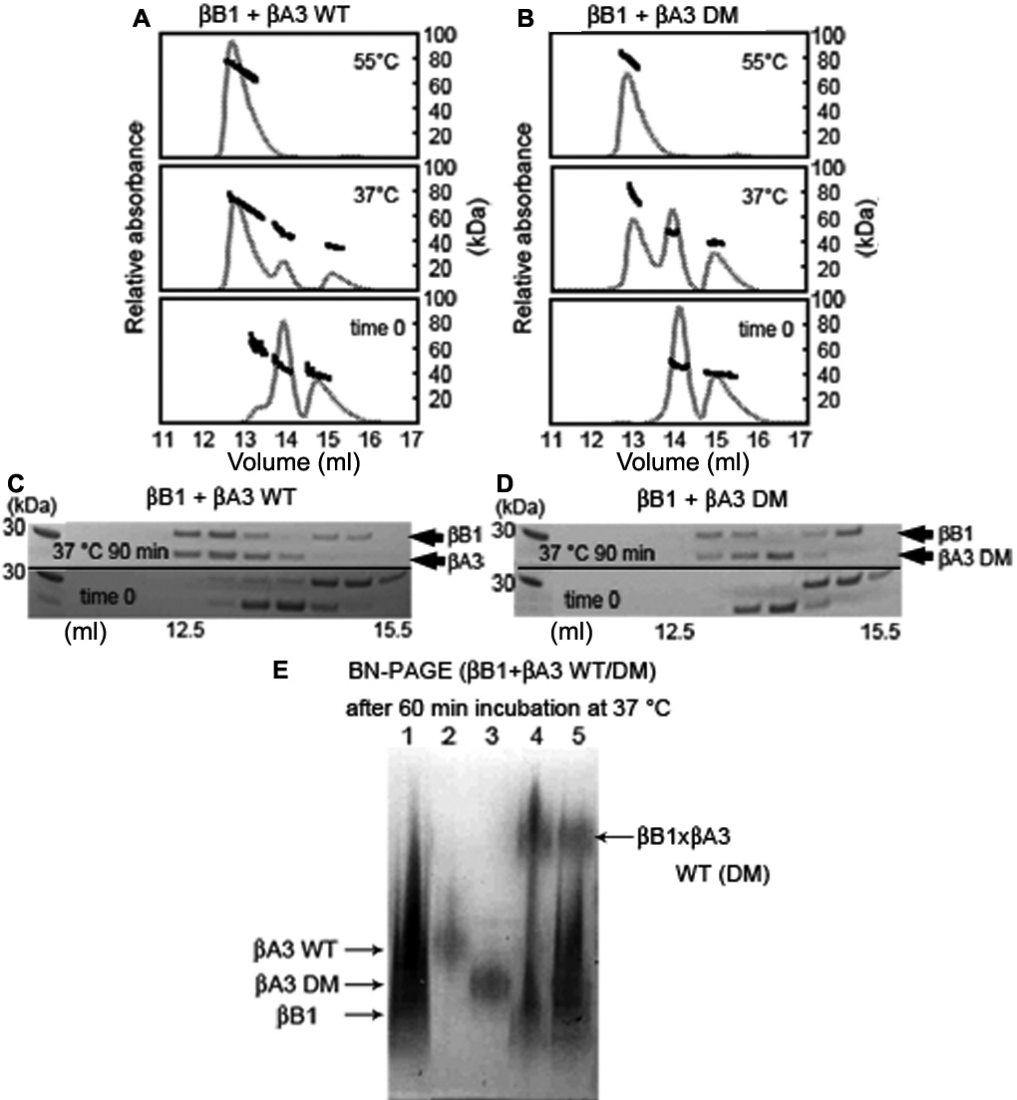
Hetero-oligomerization of βB1 with βA3. Similar size oligomers were obtained when βB1 was mixed with either βA3 WT (**A**) or βA3 DM (**B**). Mixtures were immediately subjected to SEC-MALS (bottom panel) or incubated at 37 °C (middle panel) or 55 °C (top panel) for 90 min as in Figure 2. Note the decreased amount of the βB1:βA3 DM compared to the βB1:βA3 WT complex. Eluting fractions of βB1 mixed with either βA3 WT (**C**) or βA3 DM (**D**) were collected and subjected to SDS-PAGE. Both subunits, βB1 and βA3 WT or DM, were present in the complex. Hetero-oligomer formation was confirmed by subjecting mixtures of βB1 with βA3 WT or βA3 DM incubated at 37 °C for 60 min to Blue-Native-PAGE (**E**). Proteins were βB1 (lane 1), βA3 WT (lane 2), βA3 DM (lane 3), βB1:βA3 WT (lane 4), and βB1:βA3 DM (lane 5).

Both WT βA3 and DM βA3 formed a complex with βB1 that eluted as a polydispersed peak at 37 °C (Figure 3A,B). The protein composition of the hetero-oligomer peak visualized by SDS–PAGE indicated the presence of equal amounts of each subunit suggesting predominantly hetero-tetramer formation (Figure 3C,D). The appearance of diffused high molecular weight bands of the mixed samples resolved by BN-PAGE also indicated polydispersity (Figure 3E). Complex formation increased at 55 °C, accompanied by a decrease in the amount of individual βB1 and βA3 protein peaks.

**Figure 4:**
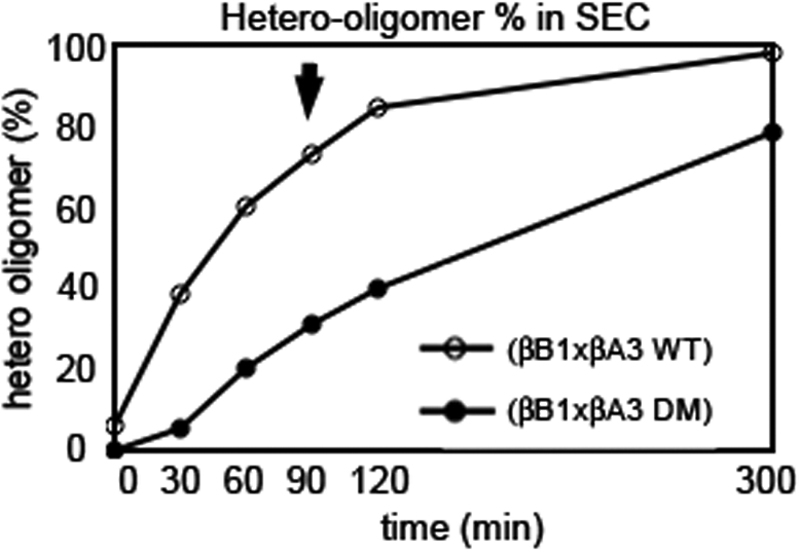
Hetero-oilgomerization of βB1 with βA3 as a function of time. βB1 was mixed with either βA3 WT (open circles) or βA3 DM (closed circles) and incubated at 37 °C from 0-300 min and then subjected to SEC-MALS. The relative amount of complex was determined from the peak area. Formation of the βB1:βA3 DM complex was slower than formation of the βB1:βA3 WT complex. Arrow indicates 90 min time point showing 40 % difference between WT and DM.

Next, βB1 was mixed with WT βA3 or DM βA3 at 37 °C as a function of time (Figure 4). Immediately upon mixing βB1 with WT βA3, a higher-ordered peak was detected by SEC-MALS, not present with DM βA3. While, the total amount of the βB1-WT βA3 complex and the βB1-DM βA3 complex were nearly the same after 300 min incubation, the βB1-DM βA3 complex formation was slower (Figure 4). Approximately 40% less βB1-DM βA3 complex formed than the βB1-WT βA3 at 37 °C after 90 min.

**Figure 5:**
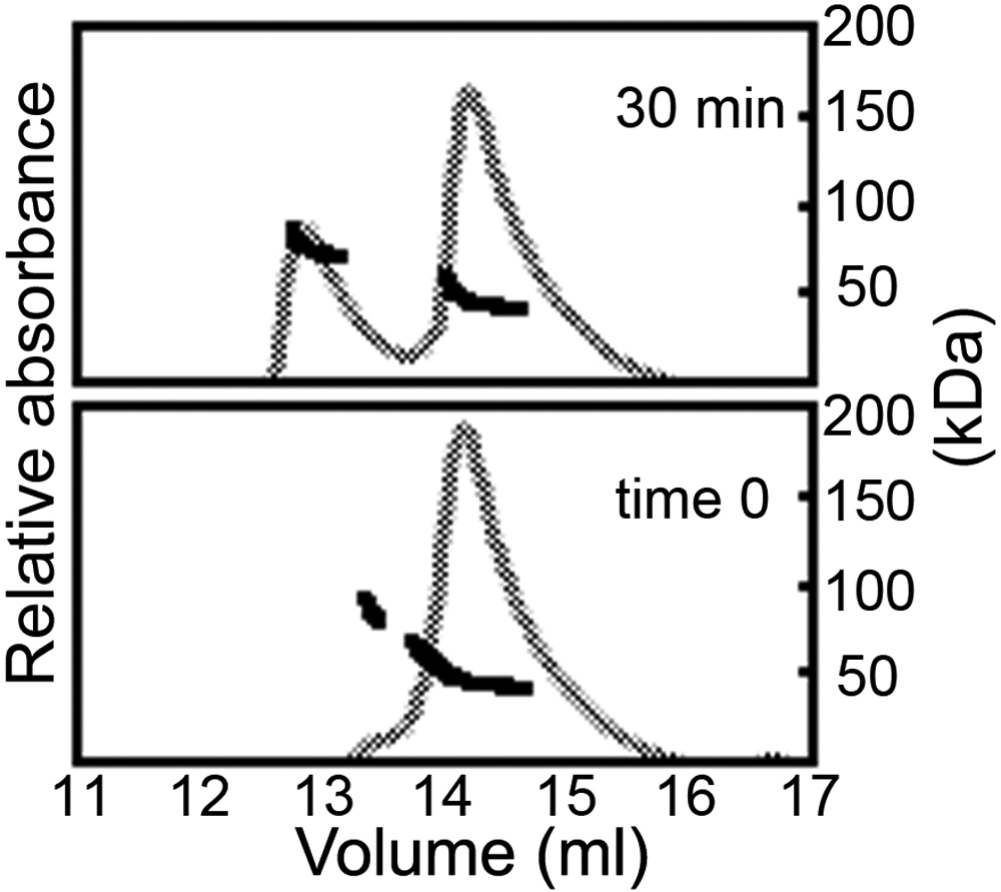
Hetero-oligomerization of βB1 dimers with βA3 dimers. In order to mix βB1 dimers with βA3 dimers, the concentration of βB1 was increased to favor βB1 dimer formation. A 5:1 molar ratio of βB1:βA3 was mixed and immediately subjected to SEC-MALS (bottom panel) or after 30 min at 37 °C (top panel). Molar masses are indicated by the closed squares and the UV absorbance at 280 nm by the grey tracing. Similar molar masses are obtained for the hetero-oligomer as in Figure 3.

βB1 was a mixture of monomer and dimers at the protein concentrations used (Figure 2A). To mix a dimer βB1 with a dimer βA3, experiments were repeated with a 5:1 ratio of βB1 to βA3 (Figure 5). Both proteins were present as dimers as detected by the overlapping peaks in SEC-MALS at 0 min. After a 30 min incubation at 37 °C, a hetero-oligomer peak eluted at the same position as the 1:1 protein peak (Figure 5 and Figure 3A). This suggested that dimers associated to form the hetero-oligomers.

### Hetero-oligomerization of βA3 with βB2

**Figure 6:**
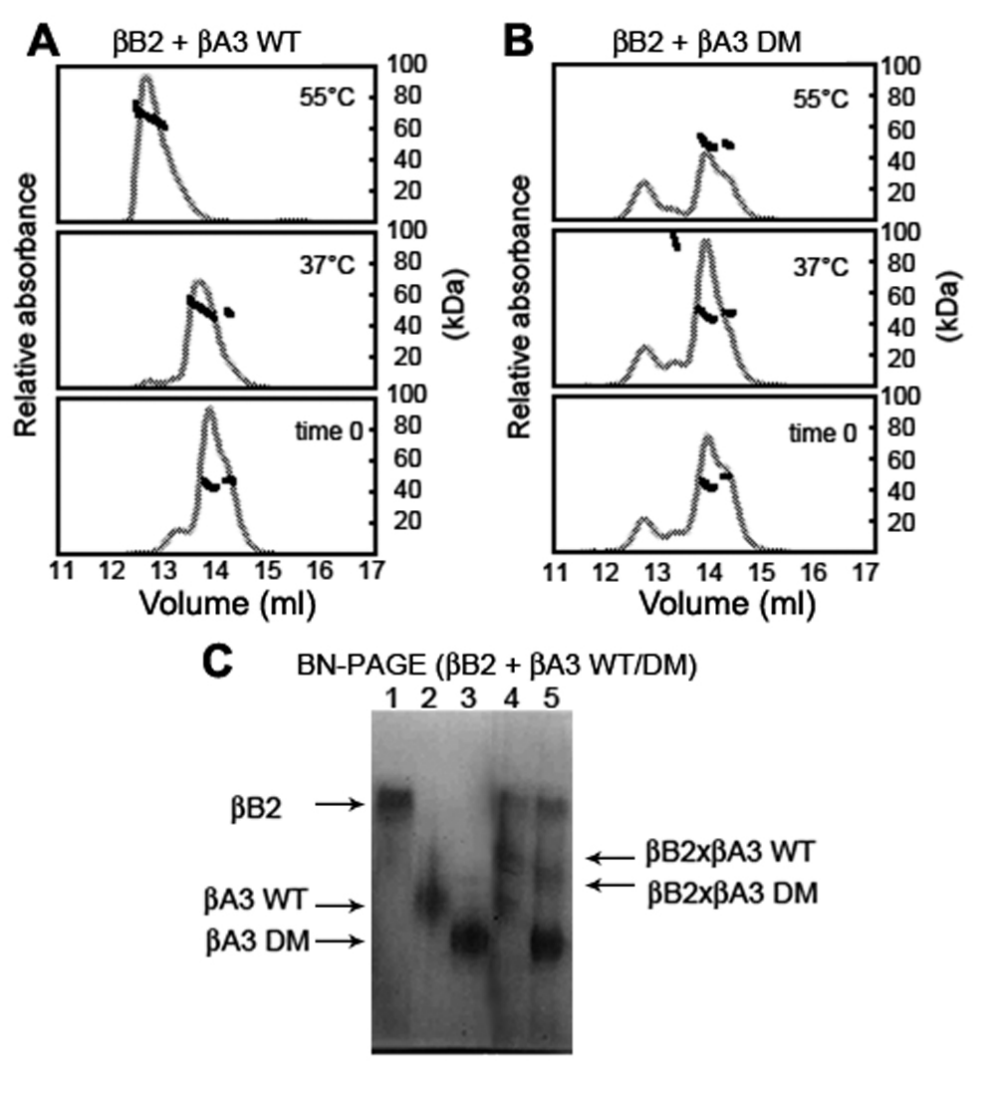
Hetero-oligomerization of βB2 with βA3. βB2 was mixed with either βA3 WT (**A**) or βA3 DM (**B**) and mixtures were immediately subjected to SEC-MALS (bottom panel) or incubated at 37 °C (middle panel) or 55 °C (top panel) for 90 min as for βB1 in Figure 3. Molar masses of βB2:βA3 WT peaks indicated predominantly dimers at 37 °C with a slight shift in elution volume and oligomers at 55 °C. Molar masses of βB2:βA3 DM peaks indicated predominantly dimers at 37 °C without a shift in elution volume and no oligomers were detected at 55 °C. Differences in hetero-oligomer formation were confirmed by subjecting mixtures of βB2 with βA3 WT or βA3 DM incubated at 37 °C for 60 min to Blue-Native-PAGE (**C**). Proteins were βB2 (lane 1), βA3 WT (lane 2), βA3 DM (lane 3), βB2:βA3 WT (lane 4), and βB2:βA3 DM (lane 5). Arrows indicate migration position of proteins.

The βB2-βA3 complex differed from that of the βB1-βA3 complex and was predominantly a hetero-dimer at 37 °C. βB2 and βA3 subunits differed only slightly in their elution (Figure 2B,C), with the shoulder present in Figure 6 attributed to βB2. After 90 min at 37 °C, there was a slight shift in the main peak partly masking the βB2 tetramer peak. The molar mass of 53-57 kDa at the peak suggested hetero-dimer formation (Figure 6A). After 90 min at 55 °C, there was a further shift in the WT βB2-βA3 peak, with a molar mass from 67 to 73 kDa (Figure 6A) that suggested a higher-ordered oligomer similar to the βB1-βA3 hetero-oligomer.

In contrast to WT βA3, DM βA3 did not readily form a complex with βB2 that was detected by SEC-MALS (Figure 6B). After 90 min at 37 °C, the shoulder disappeared with an increase in peak height. However, there was no shift in elution of the peak and the leading edge of the peak was at 55 kDa. After 90 min at 55 °C, there was also not a shift in elution of the peak and the late eluting βB2 shoulder was still present. Of note was the higher-ordered oligomer peak present immediately upon mixing βB2 and DM βA3 (Figure 6B). This peak did not increase with time or temperature and may represent a subpopulation of readily formed aggregate. Upon heating at 55 °C, there was a 50% decrease in the height of the main peak and precipitate was visible in the sample.

Because of the close molecular weights of βB2 and βA3, proteins were analyzed by BN-PAGE instead of SDS–PAGE (Figure 6C). WT βA3 and DM βA3 also migrated differently during BN-PAGE and suggested the two proteins had different shapes. After 60 min preincubation, samples were resolved by BN-PAGE for a minimum of 4 h at 4 °C. Diffused protein bands were visualized between βB2 and βA3 indicating a hetero-oligomer had formed, with less of the βB2-DM βA3 forming (Figure 6C). During the long analysis time for BN-PAGE, proteins may have formed complexes not detected during SEC-MALS.

### Effects of deamidation on the hetero-oligomer structure

**Figure 7:**
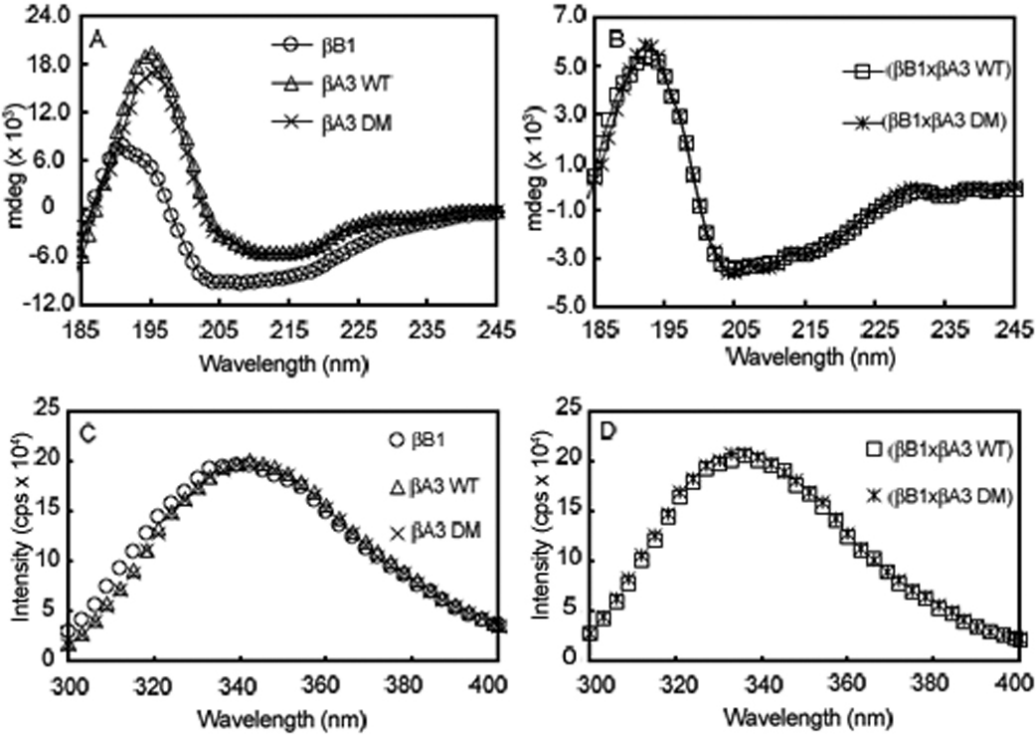
Structures of hetero-oligomer complexes. Eluting protein peaks from SEC-MALS were further analyzed by far-UV CD (**A**, **B**) and fluorescence spectrometry with excitation at 283 nm (**C**, **D**). Data were obtained of individual β-subunits (**A**, **C**) and complexes of βB1:βA3 WT or DM (**B**, **D**) and indicated similar structure of the the βB1:βA3 WT and βB1:βA3 DM complexes.

Circular dichroism measurements indicated different secondary structures for βB1 and βA3, with little difference between WT βA3 and DM βA3 as previously shown (Figure 7 and [10,27]). Circular dichroism spectra were also similar for complexes of either βB1 and WT βA3 or βB1 and DM βA3. Both hetero-oligomer complexes displayed secondary structure with maxima at 192 nm and minima at 204 nm. A slight minima at 235 nm was also observed, characteristic of βA3 [22]. The minima at 204 reflected the contribution of βB1 [10].

βA3 has nine tryptophans with five buried and βB1 has eight tryptophans with four buried, resulting in similar fluorescence spectra (Figure 7 and [22]). Upon hetero-oligomer formation there was a slight blue-shift in the fluorescence peak to 335 nm (Figure 7). Emission spectra for these proteins were the same at either 283 nm or 293 nm. The higher-ordered structures were similar for the βB1-WT βA3 or βB1-DM βA3 hetero-oligomers.

### βB1, but not βB2 prevented thermal aggregation of deamidated βA3

**Figure 8:**
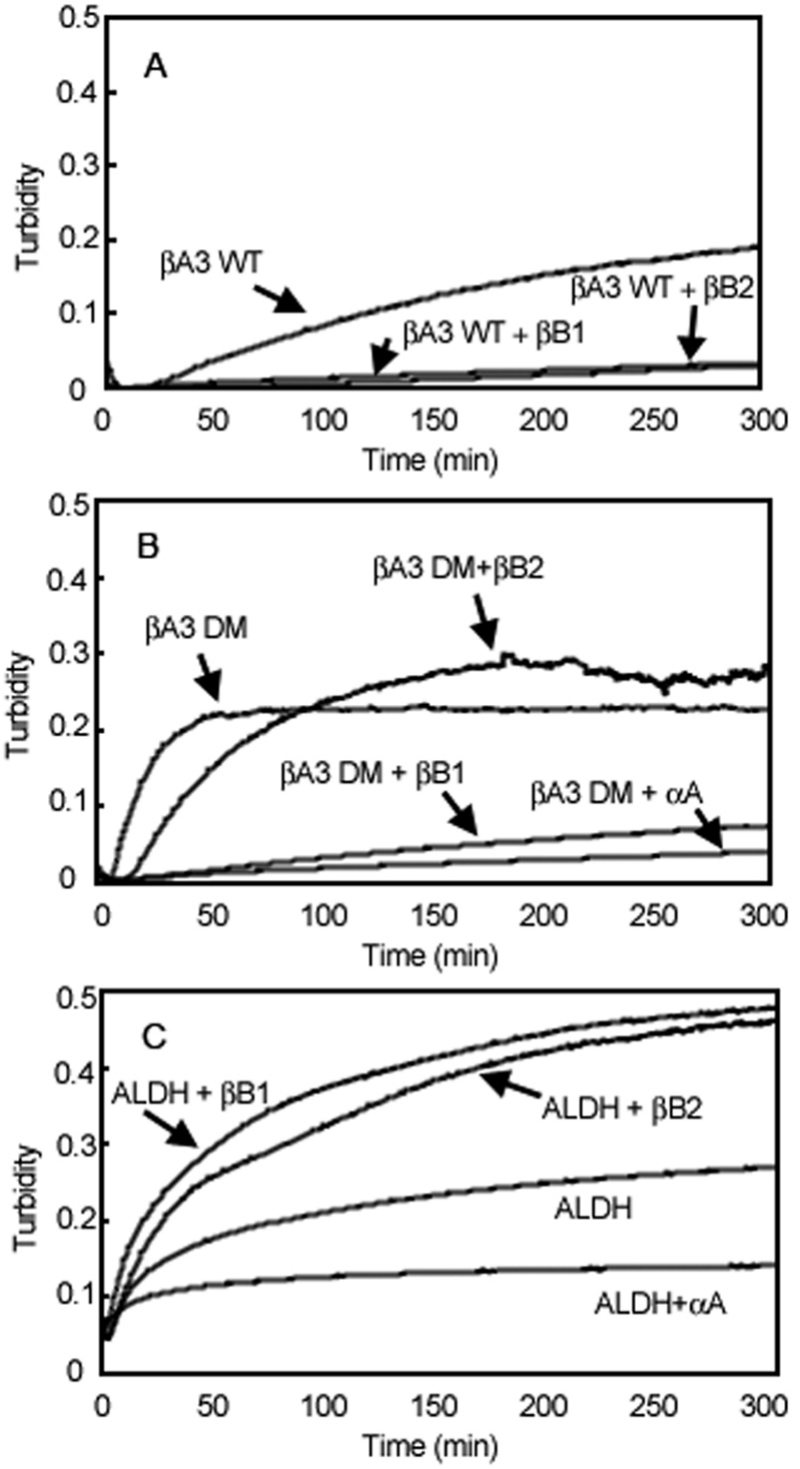
Thermal stability of β-crystallin complexes. Samples were heated at 55 °C and the turbidity of the solutions measured at 405 nm. βB1- and βB2-crystallin were mixed with βA3 WT (**A**) or βA3 DM (**B**). As a control, βA3 DM was also mixed with the known chaperone, αA-crystallin (**B**). In order to determine if either βB1 or βB2 had chaperone-like activity, the substrate alcohol dehydrogenase (ALDH) was used (**C**). Both βB1 and βB2 decreased the heat induced precipitation of βA3 WT, but only βB1 decreased the precipitation of βA3 DM. Neither βB1 nor βB2 prevented the precipitation of ALDH.

To investigate the stability of βB1-βA3 hetero-oligomers, samples were subjected to heat denaturation. Both βB1 and βB2 prevented the precipitation of WT βA3 (Figure 8A). A major finding of the present study was that βB1, but not βB2 was able to prevent heat induced precipitation of DM βA3, similar to prevention by the chaperone, αA-crystallin (Figure 8B). As a control, the model substrate, ALDH, aggregated during heating, which was prevented by αA-crystallin, but not βB1 or βB2 crystallins (Figure 8C).

## Discussion

The major findings of this study were: 1) deamidation in βA3 decreased its interaction with other β-crystallin subunits, 2) during thermal denaturation of deamidated βA3, βB1 was able to prevent precipitation, and 3) the predicted interface in the βA3 dimer may also be important in the βB1 hetero-oligomer. These results suggest that deamidation contributes to cataract formation by disrupting hetero-oligomer interactions.

### Deamidation decreases βA3 oligomerization with other β- crystallin subunits

Deamidation at the predicted interacting interface in the βA3-dimer decreased the hetero-oligomer formation of βA3 with either βB1 or βB2. This is significant because deamidation may disrupt higher-ordered oligomers necessary for lens transparency and contribute to the accumulation of deamidated crystallins in the insoluble proteins observed in aged and cataractous lenses. While much is known about the effects of post-translational modifications on homo-oligomers, little is known about the effects on the more relevant hetero-oligomer interactions. We have previously reported that deamidations on the surface of βA3 altered its oligomerization with βB1 dependent on the site [28]. This paper further supports that deamidation can disrupt oligomer formation.

### βB1 stabilizes deamidated βA3

βB1, by forming a hetero-oligomer with DM βA3, prevented the heat induced precipitation of DM βA3. In the absence of βB1, DM βA3 rapidly precipitated at 55 °C. At the concentrations used, βA3 dimers were mixed with βB1 monomers and dimers that then formed hetero-oligomers. Because deamidated βA3 crystallin was not heat stable alone, the increased stability, can be attributed to the formation of the interactions with βB1 crystallin in the hetero-oligomer.

A stabilizing affect was not seen with βB2, as would be expected since βB2 did not form a hetero-oligomer with DM βA3 at 55 °C. Even though βB2 is more heat stable than DM βA3, it was not able to prevent the precipitation of DM βA3. In contrast, both βB1 and βB2 were able to prevent the thermal denaturation of WT βA3. βB1 appears to be a better “solubilizing partner” than βB2. Because βB1 did not prevent the precipitation of ALDH, it’s stabilizing properties are most likely related to its ability to subunit exchange with the βA3 homo-dimer.

### Interactions between β-crystallin subunits

Both WT βA3 and deamidated βA3 formed tetramers with βB1. The molar masses and asymmetric shape of the eluted βB1-βA3 hetero-oligomer peak during SEC-MALS suggested a mixture of hetero-tetramers and hetero-dimers, as has previously been reported [22,28]. The βB1-WT βA3 and βB1-DM βA3 hetero-oligomers migrated similarly on BN-PAGE suggesting similar shapes. Far CD and fluorescence analysis also indicated the hetero-oligomers had similar secondary and tertiary structures.

The predicted structures of βB1 and βB2 from crystallography are known and differ [17,18], while the structure of βA3 is not known. Of interest, then, is that the CD spectra of the βB1-βA3 hetero-oligomer more closely matched that of βB1 with minima at both 205 and 218 nm. Since, truncated βB1 shows a single minimum at 218 nm [24], the double minima of the βB1-βA3 complex reflects the contribution of the long NH_2_-terminal extension of βB1. It cannot be said from these data if the complex also has a bent linker as does the βB1 homo-dimer.

We have previously reported structural changes in DM βA3 [27], which was further supported here by its differing migration from WT βA3 on BN-PAGE. Deamidation by introducing a negative charge at the predicted hydrophobic interface in βA3 disrupted its structure and decreased interaction with βB1without completely preventing formation of a mixed oligomer. Therefore, deamidation at the predicted interface in the βA3 dimer is also important in the formation of the βA3-βB1 hetero-oligomer.

In contrast to βB1, βB2 formed a hetero-dimer with WT βA3 or DM βA3. The hetero-dimer migrated on BN-PAGE as a faint band between the βB2 dimer and βA3 dimer, suggesting the shape was less elongated than the βB2 extended dimer. There was also a slight difference in migration between the βB2-WT βA3 and βB2-DM βA3 dimers, suggesting different shapes of the mixed complexes.

In summary, deamidation decreased formation of hetero-oligomers between β-crystallin subunits with specific β-crystallin interactions protective against insolubilization. An excess accumulation of deamidated β-crystallins in vivo may disrupt normal protein–protein interactions and diminish the stabilizing effects between them, thus, contributing to the accumulation of insoluble β-crystallins during aging and cataracts.
